# Control of the Immune Response by Pro-Angiogenic Factors

**DOI:** 10.3389/fonc.2014.00070

**Published:** 2014-04-02

**Authors:** Thibault Voron, Elie Marcheteau, Simon Pernot, Orianne Colussi, Eric Tartour, Julien Taieb, Magali Terme

**Affiliations:** ^1^INSERM U970, PARCC (Paris Cardiovascular Research Center), Université Paris-Descartes, Sorbonne Paris Cité, Paris, France; ^2^Service d’Hépatogastroentérologie et d’Oncologie Digestive, Hôpital Européen Georges Pompidou, Paris, France; ^3^Service d’Immunologie Biologique, Hôpital Européen Georges Pompidou, Paris, France

**Keywords:** pro-angiogenic factors, VEGF-A, PlGF, tumor, immunosuppression, regulatory T cells, MDSC, immunotherapy

## Abstract

The progressive conversion of normal cells into cancer cells is characterized by the acquisition of eight hallmarks. Among these criteria, the capability of the cancer cell to avoid the immune destruction has been noted. Thus, tumors develop mechanisms to become invisible to the immune system, such as the induction of immunosuppressive cells, which are able to inhibit the development of an efficient immune response. Molecules produced in the tumor microenvironment are involved in the occurrence of an immunosuppressive microenvironment. Recently, it has been shown that vascular endothelial growth factor A (VEGF-A) exhibits immunosuppressive properties in addition to its pro-angiogenic activities. VEGF-A can induce the accumulation of immature dendritic cells, myeloid-derived suppressor cells, regulatory T cells, and inhibit the migration of T lymphocytes to the tumor. Other pro-angiogenic factors such as placental growth factor (PlGF) could also participate in tumor-induced immunosuppression, but only few works have been performed on this point. Here, we review the impact of pro-angiogenic factors (especially VEGF-A) on immune cells. Anti-angiogenic molecules, which target VEGF-A/VEGFR axis, have been developed in the last decades and are commonly used to treat cancer patients. These drugs have anti-angiogenic properties but can also counteract the tumor-induced immunosuppression. Based on these immunomodulatory properties, anti-angiogenic molecules could be efficiently associated with immunotherapeutic strategies in preclinical models. These combinations are currently under investigation in cancer patients.

## Introduction

Tumorigenesis is a multistep process in which a succession of genetic alterations, conferring some proliferative advantages, leads to the progressive conversion of normal cells into cancer cells. In 2000, Hanahan and Weinberg have grouped these genetic alterations into six distinctive and complementary biologic capabilities that constitute the six hallmarks criteria of cancer (1). They include sustaining proliferative signaling, evading growth suppressors, resisting cell death, enabling replicative immortality, activating invasion and metastasis, and inducing angiogenesis. Although these hallmarks criteria are mainly due to the accumulation of cell-intrinsic modifications, emerging evidences suggest that “tumor microenvironment,” i.e., cells infiltrating the tumors and molecules produced inside, contributes also to the biology of many cancers. In the light of this new concept, Hanahan and Weinberg have revisited their criteria in 2011 and added two emerging criteria: deregulating cellular energetics and avoiding immune destruction (2).

The concept that the immune system can recognize and destroy cancer cells and so repress the development of tumor has first been described in 1909 by Paul Erhlich and then rephrased in 1957 by Sir MacFarlane Burnet and Lewis Thomas in the cancer immunosurveillance hypothesis (3–5). Nevertheless, this hypothesis was definitively accepted recently with studies highlighting the role of the immune system in controlling cancer development in animal models and in immunodeficient or immunosuppressed patients. Thus, Shankaran et al. have demonstrated that mice deficient for T and B lymphocytes (RAG2^−/−^ mice) or mice deficient for interferon gamma signaling develop more frequently spontaneous cancer and carcinogen-induced cancer than wild type mice (6). In humans, immunodeficient or immunosuppressed patients had a higher incidence of cancer of non-viral origin (colon, lung, pancreas, melanoma) than immunocompetent patients (7–10). In addition, some immunosuppressed transplant recipients have been observed to develop tumor derived from the donor organ, underlining the importance of the immune system as an effective barrier to the tumor progression (11, 12). Finally, recent works have demonstrated that tumor infiltration by different immune cells (NK or T cells) was correlated with good prognosis in various cancers (13). For example, in colorectal cancer, Pagès and Galon have demonstrated that the absence of pathological signs of early metastatic invasion (vascular emboli, lymphatic invasion, and perineural invasion) was correlated with the presence of effector memory T cells (CD45RO^+^) within the tumor and a better overall and disease-free survival (14). The density of T cells (CD3^+^) infiltrating the tumor was also correlated with the outcome and seems to have a better and independent prognostic value for overall survival than the usual histopathologic prognostic factor (UICC-TNM classification) (15). Thus, to become clinically detectable in the immunocompetent host, cancer cells have to bypass this immunosurveillance by downregulating expression of molecules that are involved in immune recognition or by engendering a state of immunosuppression linked to the recruitment of immunoregulatory cells within the tumor or the production of immunosuppressive factors.

Recently, accumulative evidence suggests that pro-angiogenic factors could induce tumor growth and metastasis, not only by promoting angiogenesis but also by favoring this immunosuppressive microenvironment.

A better knowledge of this link between angiogenesis and immune escape could lead investigators to devise new therapeutic strategies combining anti-angiogenic therapy and immunotherapy.

## Pro-Angiogenic Factors Promote Intratumoral Immunosuppressive Microenvironment

**Figure 1:**
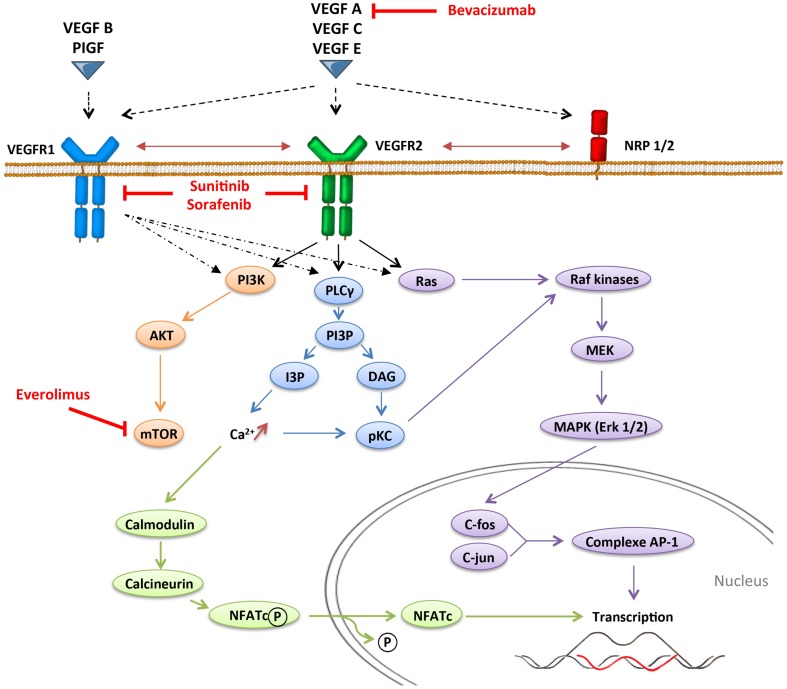
**VEGFR signaling pathways**.

Angiogenesis, which is defined by the sprouting of new vessels from pre-existing ones, is a dynamic process that is observed under physiological conditions (embryogenesis and wound healing) but also under pathological conditions as tumor progression. In contrast with physiological angiogenesis that is transiently activated, pathological angiogenesis is almost always activated and remains on, resulting from a permanent imbalance between pro-angiogenic and anti-angiogenic factors. The absence of oxygen in the center of the tumor induces a hypoxic stress, which plays a key role in the regulation of angiogenesis. Hypoxia induces the stabilization and nuclear accumulation of the hypoxia-inducible factor (HIF), transcriptional factors, which results in the production of many pro-angiogenic factors including vascular endothelial growth factor A (VEGF-A) (16). VEGF-A plays a central role in inducing tumor angiogenesis. VEGF-A is a glycoprotein (45 kDa) that is produced by nearly all tumor cells (17) and is found at elevated levels in the serum of cancer patients (18). It binds to two key receptors, VEGFR1 (Flt-1) and VEGFR2 (Flk-1, KDR), and one co-receptor (Neuropilin-1) to exert its pro-angiogenic activities. Although VEGF-A was initially identified as an endothelial cell-specific growth factor, it has become increasingly apparent that the functions of VEGF-A were more extensive, and especially act on immunity. Other pro-angiogenic factors like placental growth factor (PlGF) can also modulate intratumoral immunosuppressive microenvironment. PlGF is a member of the VEGF family, which is produced by tumor cells and stromal cells. It binds to VEGFR1 and induces migration and maturation of blood vessels by favoring proliferation, migration, and survival of endothelial cells. PlGF is correlated to tumor stage, to metastatic invasion, and inversely to survival in different solid tumors (19). PlGF also exhibits immunomodulatory properties (20). VEGF receptors can be expressed on endothelial cells, tumor cells, and some immune cells (21–23). VEGFR1 binds VEGF-A with higher affinity than VEGFR2 does, but VEGFR1 has a poor tyrosine kinase activity. Signaling pathways involved in VEGFR1 are confused. VEGFR1 may form heterodimers with VEGFR2 on endothelial cells as a consequence of VEGF-A binding and may also function as a decoy receptor sequestering VEGF-A from VEGFR2. But VEGFR1 may also transduce signals resulting in activation of proliferation, migration of the cells by activating Erk/MAPkinases, PI3K/Akt, PLCγ, and p38/MAPkinases (Figure 1) (24). VEGFR2 activation induces many biological responses such as proliferation, migration, survival by activation of PLCγ, Raf-kinases, and PI3K pathways (Figure 1).

### VEGF and PlGF inhibit dendritic cell maturation

Mature DCs are critical elements of anti-tumor immunity since professional antigen-presenting cells (APC) are responsible for the presentation of tumor-specific antigens and the triggering of an adaptive immune response mediated by T-cells (25). Conversely, in tumor-bearing mice (26) and cancer patients, differentiation and maturation of DC are impaired. Thus, immature DCs are not able to efficiently present tumor antigens to naive T-cells and therefore promote immune tolerance. The increase of the proportion of immature DC in the peripheral blood is correlated with the stage of the disease in cancer patients and is partially corrected by surgery, suggesting that this phenomenon is linked to a tumor-derived factor (27). The proportion of immature DC in the blood of cancer patients is closely associated with an increased VEGF-A plasma level. In mouse models, different studies have shown that VEGF-A binding to VEGFR1 blocks the activation of the transcriptional factor NF-κB (nuclear factor-κB) and leads to inhibit DC maturation (28, 29). According with these results, PlGF, which binds specifically to VEGFR1, can also modulate DC differentiation, through the same mechanism (20, 30). In an *in vitro* model of dendritic cell differentiation from embryonic stem cells exposed to VEGF-A, Dikov et al. showed that VEGFR1 is involved in the inhibition of the final maturation of DC and VEGFR2 affects the differentiation of DC from early hematopoietic progenitors (20). Another *in vitro* study has shown that VEGF-A can alter the differentiation of monocytes into DC, effect reversed by anti-VEGF-A (bevacizumab) or sorafenib, an anti-angiogenic molecule targeting different receptors (VEGFR, PDGFR, and Raf-kinases) (31). Administration of exogenous VEGF-A to tumor-free mice using osmotic pumps to mimic the VEGF concentrations observed in advanced cancer patients also blocks the ability of DC to stimulate allogeneic T-cell proliferation (32). Altogether, these results provide strong evidence that pro-angiogenic factor can inhibit DC maturation through both VEGFR1 and VEGFR2 pathways.

### Pro-angiogenic factors favor the accumulation of immunoregulatory cells (MDSC, Treg, tumor-associated macrophages, Tie-2^+^ monocytes)

Myeloid-derived suppressor cells (MDSC) are a heterogeneous group of cells of myeloid origin, including myeloid progenitor cells and immature myeloid cells (macrophages, granulocytes, and dendritic cells) with immunosuppressive properties. MDSC accumulation in the tumor microenvironment leads to suppress T-cell response in different ways. MDSCs can first metabolize l-Arginine, an essential amino-acid for adult mammals that is required for T-cell proliferation (33, 34): (i) using Arginase1, which results in a reduction of extra-cellular levels of l-Arginine (35); (ii) using the iNOS enzyme, which results in the generation of NO. The accumulation of NO in the tumor microenvironment blocks the proliferation of T cells and induces their apoptosis resulting in a decrease of tumor-infiltrating T-cells (36, 37). MDSCs can also exert their immunosuppressive properties by producing indoleamine 2,3-dioxygenase, reactive oxygen species (ROS), like radical superoxide (O^2^•−) (38). Finally, reactivity between radical superoxide (O^2^•−) and NO, both produced by MDSC, leads to the formation of free radical peroxynitrite in the tumoral microenvironment that blocks the ability of T cells to recognize specific peptide/MHC complexes and perform their anti-tumor activity. MDSC can also control NK cell activation through membrane-bound TGFβ and NKp30 in an orthotopic mouse model of liver cancer and in hepatocellular carcinoma-bearing mice, respectively (39, 40). VEGF-A can promote the accumulation of MDSC (41). Indeed, Almand et al. reported an increase of MDSC in cancer patients, that is, associated with a decrease of mature DC. This accumulation is correlated with the disease stage and serum VEGF-A levels (27, 42). Moreover, an increase of Gr1^+^CD11b^+^ cells (MDSC) in the spleen of tumor-free mice treated with VEGF-A compared with control mice has been observed, and this effect is mediated by VEGFR2 (32) and activation of JAK2 (JAnus Kinase 2) and the transcription factor STAT3 (signal transducer and activator of transcription 3) downstream (43).

Pro-angiogenic factors could also contribute to other immunosuppressive cell accumulation such as regulatory T cell (Treg) in tumor-bearing hosts through direct or indirect mechanisms. Thus, MDSC, which are enhanced by VEGF, could induce *de novo* development of other immunosuppressive cells as of Foxp3^+^ Tregs through a TGF-β-dependent (44, 45) and/or independent pathway (46). Thus, in a mouse model of colon carcinoma, Gr1^+^CD115^+^ MDSC were shown to mediate the development of Treg by producing IL-10 and TGF-β (44). Immature DC can also induce Treg differentiation and proliferation in a TGFβ-dependent manner (47). Moreover, we have recently shown that VEGF-A, could also directly induce Treg proliferation in tumor-bearing mice and metastatic colorectal patients in a VEGFR2-dependent manner (22). Analysis of VEGFR expression on Tregs shows that Tregs express VEGFR2 only in tumor-bearing hosts but not in tumor-free mice (22).

Tumor-associated macrophages (TAM), which are correlated with poor prognosis (48–51), are induced by VEGF-A. But its development also needs the action of other cytokines produced by the tumor such as IL-10 and IL-4 (52). Another pro-angiogenic factor, angiopoietin-2 has also an impact on immune cells. Angiopoietin-2 induces the release of IL-10 by Tie-2-expressing monocytes. Expression of angiopoietin-2 by tumor cells induces the recruitment of Tie-2-expressing monocytes in the tumor and the release of IL-10 by the cells (53, 54). IL-10 production by Tie-2-expressing monocytes suppresses T-cell proliferation, increases the ratio of CD4^+^ T cells to CD8^+^ T cells, and promotes the expansion of CD4^+^CD25^hi^FOXP3^+^ Tregs (53).

**Figure 2:**
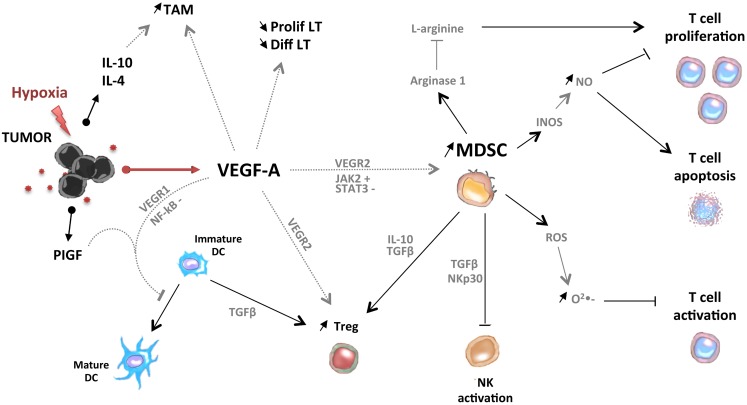
**Pro-angiogenic factors induce the development of an immunosuppressive state in tumors**. VEGF-A induces the accumulation of MDSC, immature DC, Treg, and tumor-associated macrophages (TAM). MDSC and Treg are able to control activation of T cells and NK cells.

In conclusion, pro-angiogenic factors could contribute to the accumulation of immunosuppressive cells (MDSC, Treg, TAM, and Tie-2-expressing monocytes) in tumor-bearing hosts through direct or indirect mechanisms (Figure 2).

### Pro-angiogenic factors and conventional T cells

Vascular endothelial growth factor A could inhibit the production of T cells, which are the major immune effector cells, by interfering with their development in thymus, as described by Ohm et al. (55). In this study, administration of exogenous VEGF-A to tumor-bearing mice at concentrations similar to those observed in advanced stage cancer patients leads to profound thymic atrophy, as observed in childhood malignancies, with a decrease in thymocyte cellularity. This inhibition of thymocyte maturation is caused through VEGFR2 pathway (32).

There are evidences for circulating T-cells in the periphery of tumor-bearing mice (56, 57) and cancer patients (58, 59), which are yet unable to control tumors (60). Thus, tumor angiogenesis, driven by pro-angiogenic factors, leads to the formation of a new vasculature that is structurally and functionally abnormal. New vessels are dilated, tortuous, and saccular, with disorganized and heterogeneous interconnections, resulting in hyperpermeable and insufficient vessels that could contribute to impediment of T cells extravasation. This hypothesis is reinforced with the recent work of Hamzah et al. using a genetically modified model of spontaneous pancreatic islet carcinoma in which the inactivation of Rgs5 (Regulator of G-protein signaling 5), a master gene controlling the aberrant morphology of tumor vasculature in mice and expressed by pericytes in the vascular bed, leads to normalization of tumors vessels (61). In this model where Rgs5 is inactivated, adoptive T-cell transfer results in massive infiltration of CD8^+^ and CD4^+^ lymphocytes within the tumor, whereas wild type tumors showed no significant increase of intratumoral immune cells. In another model, overexpression of the histidine-rich glycoprotein (HRG) induces tumor vessels normalization through a down regulation of PlGF, and at the same time, leads to an increase of CD8^+^ lymphocyte infiltration within the tumor (62). Taken together, these results show a direct link between tumor vasculature normalization and enhanced immune cell infiltration.

Vascular endothelial growth factor A has also been shown to decrease effector functions (proliferation and cytotoxicity) of T lymphocytes obtained from peripheral blood and ascites of ovarian cancer patients (63, 64).

On the other hand, exposure of tumor cells to hypoxia prevents cytotoxic T lymphocyte (CTL)-mediated lysis of tumor cells (65). This phenomenon depends on hypoxia-induced VEGF-A since VEGF-A neutralization restores the susceptibility of tumor cells to CTL lysis and also on STAT3 activation.

Thus, VEGF-A can induce an immunosuppressive microenvironment by targeting immune cells but also tumor cells.

## Anti-Angiogenic Therapy Can Reverse Immunosuppression

Anti-angiogenic molecules have been developed to inhibit a major hallmark of cancer: angiogenesis. These treatments target preferentially the VEGF pathway since it has been revealed as a key regulator of angiogenesis, and include three types of VEGF-targeted agents: neutralizing antibodies to VEGF or VEGF receptors (like bevacizumab), tyrosine kinase inhibitors (TKI) that block intracellular signaling pathway (like sunitinib that targets VEGFR1–3, PDGFR, c-kit, and Flt3, or sorafenib that targets VEGFR1–3, PDGFR, c-kit, and Raf-kinases), and inhibitors of the mTOR pathway (like temsirolimus and everolimus) (Figure 1). The use of this VEGF-targeted therapy has been approved for metastatic colorectal cancer (66), hepatocellular carcinoma (67), clear-cell renal carcinoma (68), breast cancer (69), and non-small-cell lung carcinoma (70), alone or in combination with chemotherapy. These treatments can impact multiple pathways, act on tumor and endothelial cells, and block the neoangiogenesis. But they can also modulate other cells especially cells expressing VEGFR. According with the results presented above on the impact of pro-angiogenic factors on immunity, recent data suggest that anti-angiogenic therapy could reverse some immunosuppressive mechanisms involved in tumor escape and tumor growth and lead to improve cancer immunosurveillance.

### VEGF-targeted therapy modulates number and functions of immunosuppressive cells

Gabrilovich et al. have shown that anti-VEGF antibody significantly enhances the maturation of DC, resulting in an increase of number and functions of lymph nodes and spleen DCs in tumor-bearing mice treated with anti-VEGF-A (71). Osada has then confirmed this result in humans by demonstrating an increase in the number of DCs in peripheral blood and an improvement of DCs functions in advanced solid cancer patients treated with bevacizumab (anti-VEGF-A) (72). Blockade of VEGF-A pathway by anti-VEGF-A antibody in tumor-bearing mice also leads to a significant reduction of MDSCs in peripheral blood, as compared with untreated mice (73). Similarly, a decrease in the absolute number of MDSC in the spleen, bone marrow, and tumor in different tumor models (the MCA26 colorectal cancer, or the Renca renal cancer), has also been observed after treatment with sunitinib (74, 75). Furthermore, this decrease was associated with a reduction of immunosuppressive activity *in vitro* of MDSCs from sunitinib-treated mice, compared with MDSC from PBS-treated control mice (74). Inhibition of VEGF-A pathway could explain its activity on MDSCs since anti-VEGF antibody decreases a CD11b^+^ VEGFR1^+^ subset of MDSC that is able to suppress T-cell response (73) but Xin et al. have also demonstrated that sunitinib could act on MDSCs by inhibiting Stat3 (75). Analysis of the impact of sunitinib on MDSC reveals that it inhibits the proliferation of the monocytic subset of MDSC (Gr1^lo^) and induces the apoptosis of the granulocytic subset (Gr1^hi^) (75). Treatment with sorafenib in a mouse model of liver tumor leads also to a decrease in MDSC levels in the spleen and bone marrow (76). Since MDSC levels in the peripheral blood of patients with head and neck cancer, non-small-cell lung cancer, breast cancer are positively correlated with plasma level of VEGF-A, VEGF-targeted therapy could induce a decrease of MDSCs in peripheral blood of cancer patients (27). In metastatic renal cell carcinoma patients, treatment with sunitinib decreases the percentage of MDSCs in peripheral blood, after one cycle of treatment (5.42% of PBMC before treatment vs. 2.28% after one cycle; *p* = 0.007), but also after the second cycle (2.28 vs. 1.29%; *p* = 0.02) (77).

Vascular endothelial growth factor-targeted therapy can also decrease Treg, either by inhibiting accumulation of MDSCs and immature DC in tumor microenvironment or directly through VEGF/VEGFR pathway inhibition on Treg. Thus, we have demonstrated recently that treatment of CT26 colorectal tumor-bearing mice with sunitinib or anti-VEGF-A antibody reduces the percentage and the absolute number of Treg in the spleen and tumor as compared with non-treated tumor-bearing mice (22). In the same manner, treatment with other anti-angiogenic therapies, such as sunitinib and sorafenib, modulates accumulation of Treg in tumor and spleen of various mouse tumor models (22, 76, 78, 79). Different mechanisms of sunitinib action have been proposed: (i) Treg decrease could be associated to the reduction of MDSC (77); (ii) a blockade of conversion of conventional CD4^+^ Foxp3^−^ T cells into regulatory CD4^+^ Foxp3^+^ T cells (80); (iii) a reduction of the proliferation of pre-existing Tregs (22). In cancer patients, anti-angiogenic treatments also reduce Tregs in periphery. Sunitinib treatment decreases the number of Foxp3^+^ Tregs in the peripheral blood of metastatic renal cancer patients (79) and this decrease was positively correlated with a better overall survival (81, 82). Similarly, sorafenib treatment induces a reduction in regulatory T-cell number in the peripheral blood of patients with hepatocellular carcinoma (83). Bevacizumab, which is used in association with chemotherapy as a first-line treatment in metastatic colorectal cancer patients, also decreases regulatory T-cell proportion in the peripheral blood of these patients. This decrease is linked to a reduction of Treg proliferation (22).

### Anti-angiogenic molecules and conventional T cells

Ozao-Choy et al. have demonstrated in tumor-bearing mice that sunitinib treatment enhances the percentage and number of intratumoral CD4^+^ and CD8^+^ T-cells compared with tumor-bearing mice treated with PBS (74). Similarly, blockade of VEGF-A/VEGFR2 pathway in tumor-bearing mice improves the infiltration of adoptively transferred T cells into the tumor and tumor regression (84). This better infiltration could be associated with the capacity of anti-angiogenic molecules to normalize tumor vasculature and probably to prevent loss of ICAM-1 and VCAM-1 on endothelial cells (85). Sunitinib treatment can also modulate expression of inhibitory molecules on tumor-infiltrating T cells such as PD-1, CTLA-4 (74).

## Impact of Strategy Combining Anti-Angiogenic Therapy with Immunotherapy

Anti-angiogenic drugs have improved the treatment of many solid tumors. These molecules have anti-angiogenic impact but also immunomodulatory properties (86, 87). As described above, anti-angiogenic drugs inhibit the accumulation of immunosuppressive cells (immature myeloid cells such as immature dendritic cells and MDSC, Treg), which are able to inhibit the development of an efficient anti-tumor immune response. They can also enhance the proportion of tumor-infiltrating T lymphocytes probably by normalizing tumor vessels and by modulating the expression of adhesion molecules involved in T-lymphocyte extravasation (88). Sunitinib can also enhance the Th1 response of T lymphocytes derived from metastatic renal cancer patients after mitogen restimulation (81). However, they do not seem able to restore a spontaneous specific T-cell response to tumor antigens. Based on their immunomodulatory properties, anti-angiogenic drugs could be combined to immunotherapeutic strategies to obtain durable anti-tumor responses. Recently, immunotherapy has obtained successes, especially in the treatment of metastatic solid cancer patients, where anti-CTLA-4 or anti-PD1 antibodies administration results in an enhancement of the proportion of objective and durable responses (89, 90). Furthermore, Sipuleucel-T is the first vaccination strategy approved by the FDA for the treatment of patient with castration-resistant prostate cancer. This vaccine induces an improvement of overall survival (91). The future of immunotherapy will probably involve combination with other immunomodulatory agents. Different groups tried to combine immunotherapeutic strategies with anti-angiogenic molecules in mouse tumor models. The most commonly used molecule in these studies is sunitinib. Association of sunitinib with adenoviral vectors encoding for IL-12 and other immunostimulating molecules or pox-virus encoding for stimulatory molecules and tumor antigen decreased tumor growth in different mouse models (colorectal cancer, hepatocellular carcinoma) (74, 92). To design relevant protocols of treatment with anti-angiogenic molecules and immunotherapy, it is necessary to determine the scheduling of drug administration. Administration of sunitinib before vaccination induced a superior anti-tumor efficacy than administration after vaccination or concurrently (92). A recent work has even shown that sunitinib administration concurrently with a vaccine against a tumor antigen results in a lack of reactivity against the tumor antigen in a mouse mammary tumor model. The strategy failure seems to be due to a transient loss of CD11c^+^ CD11b^+^ APC in the lymph nodes, which inhibits the priming of T lymphocytes (93). To optimize strategies combining anti-angiogenic drugs to immunotherapy, we also need to determine which anti-angiogenic should be used and at which dosage. VEGF-A/VEGFR2 targeted therapies seem to be the best choice, since they can modulate both Treg and MDSC. Administration of low-doses of anti-VEGFR2 antibody results in a transient vascular normalization and improves the CD4^+^ and CD8^+^ tumor infiltration. Association of low-doses of anti-VEGFR2 with whole cancer cell vaccine induces anti-tumor efficacy (94).

Interestingly in metastatic renal cancer, two patients received sunitinib before radical nephrectomy and dendritic cell therapy. These two patients displayed disease stabilization after sunitinib treatment and regression of metastatic lesions after nephrectomy and DC vaccine, suggesting that sunitinib could synergize with DC vaccine (95). In an open-label phase II trial, 21 metastatic renal cancer patients received sunitinib and a DC-based vaccine (AGS-003). This vaccine consists of mature monocyte-derived DC electroporated with mRNA harvested from the patient’s tumor and synthetic CD40L mRNA. This combination resulted in an interesting progression-free survival and overall survival. An international phase 3 trial will be launched based on these results (NCT01582672). Other clinical trials are testing combination between anti-angiogenic drugs and immunotherapy in cancer patients, especially association of antibodies targeting inhibitory immune checkpoints (anti-PD-1, anti-CTLA-4) with bevacizumab or sunitinib (Table 1).

**Table 1:** **Ongoing clinical trials, according to National Cancer Institute (NCI) registration, using association of anti-angiogenic drugs with immunotherapy**.

Anti-angiogenic	Immunotherapy	Cancer	Phase	Status	Registration number
Bevacizumab	MK-3475 (anti-PD1)	Locally advanced or metastatic non-small-cell lung carcinoma	I/II	Recruiting	NCT02039674
Bevacizumab	MPDL3280A (anti-PDL1)	Advanced solid tumors	I	Recruiting	NCT01633970
Bevacizumab	Ipilimumab (anti-CTLA4)	Unresectable stage III or IV melanoma	I	Active, not recruiting	NCT00790010
Bevacizumab	Ipilimumab (Anti-CTLA4)	Unresectable stage III or IV melanoma	II	Not yet recruiting	NCT01950390
Bevacizumab	Nivolumab (anti-PD1)	Stage IIIB/IV non-small-cell lung cancer	I	Recruiting	NCT01454102
Sunitinib	Nivolumab (anti-PD1)	Metastatic renal cell carcinoma	I	Active, not recruiting	NCT01472081
Bevacizumab	Dendritic cell immunotherapy	Resected hepatic metastasis of colorectal carcinoma	II	Recruiting	NCT01348256
Bevacizumab	Vaccin TG4010	Stage IV non-small-cell lung cancer (TIME)	II/III	Recruiting	NCT01383148
Bevacizumab	Dendritic cell vaccination	Newly diagnosed or recurrent glioblastoma	I	Recruiting	NCT02010606
Sunitinib	Autologous dendritic cell immunotherapy (AGS-003)	Advanced renal cell carcinoma	III	Recruiting	NCT01582672
Sorafenib	Interleukin 21	Renal cell carcinoma	I/II	Completed (96)	NCT00389285

## Conclusion

Pro-angiogenic factors and especially VEGF-A modulate the tumor microenvironment. VEGF-A plays a key role in controlling tumor angiogenesis but also in modulating tumor-induced immunosuppression (accumulation of immature DC, MDSC, Treg). Anti-angiogenic drugs can decrease immunosuppression. However, these molecules are not able to reactivate efficient immune responses against the tumor. In this goal, association of anti-angiogenic molecules to immunotherapeutic strategies could be of major interest. Different preclinical models have shown that this combination can induce potent anti-tumor response. To optimize protocols of treatment with anti-angiogenic drugs and immunotherapeutic strategies, different parameters should be analyzed such as the scheduling of the treatment and the doses of anti-angiogenic drugs to administer.

## Conflict of Interest Statement

Simon Pernot, Julien Taieb, Magali Terme received research grant from Roche. Julien Taieb has advisory role in Roche. No potential conflicts of interest were disclosed by the other authors.
